# Computational Design of Three-Dimensional Nitrogenated Holey Graphene Structures with Phase-Dependent Optoelectronic Properties

**DOI:** 10.1021/acsomega.6c07967

**Published:** 2026-09-25

**Authors:** Jhionathan de Lima, Raphael M. Tromer, Luiz F. C. Pereira, Mauro S. Ferreira, Cristiano F. Woellner

**Affiliations:** † Department of Physics, 28122 Federal University of Parana, UFPR, Curitiba, Parana 81531-980, Brazil; ‡ Interdisciplinary Center for Science, Technology, and Innovation (CICTI), Federal University of Parana, UFPR, Curitiba, Parana 81530-000, Brazil; § Institute of Physics, University of Brasília, Brasília, Federal District 70910-900, Brazil; ∥ Center for Computational Engineering and Sciences, State University of Campinas, Campinas, Sao Paulo 13083-970, Brazil; ⊥ Physics Department, 28116 Federal University of Pernambuco Recife, Recife, Pernambuco 50670-901, Brazil; # School of Physics, 8809 Trinity College Dublin, Dublin 2 D02 PN40, Ireland; ∇ Centre for Research on Adaptive Nanostructures and Nanodevices (CRANN) and Advanced Materials and Bioengineering Research (AMBER) Centre, Trinity College Dublin, Dublin 2 D02 PN40, Ireland

## Abstract

In this work, we propose and comprehensively characterize two novel three-dimensional nitrogenated holey graphene (3D-NHG) structures, designated as the α and β phases. These architectures are predicted through a computational protocol based on the controlled compression of stacked 2D-NHG monolayers. Their dynamical and structural stabilities are assessed via phonon dispersion calculations and molecular dynamics simulations within the density-functional tight-binding framework. Density functional theory calculations reveal that the α phase is a semiconductor with an indirect HSE06 bandgap of 0.87 eV, whereas the β phase exhibits a zero-gap semiconducting character. Furthermore, optical calculations reveal strong absorption in the ultraviolet region, with negligible absorption throughout the visible range. These findings highlight the potential of 3D-NHG architectures as a versatile platform for tailoring the electronic and optical properties of carbon nitride frameworks for future optoelectronic applications.

## Graphical abstract

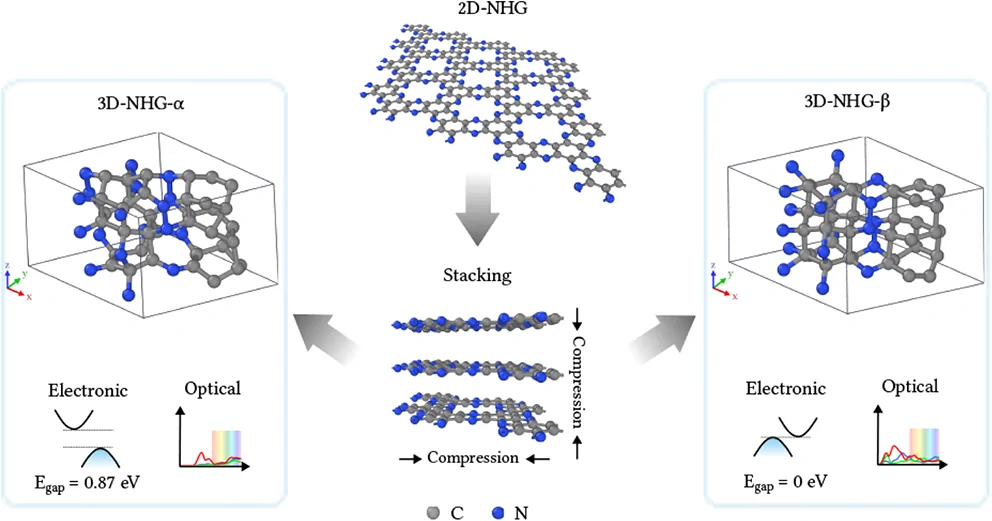

## Introduction

The search for novel carbon-based nanomaterials with tailored electronic and optical properties has been a milestone in condensed matter physics and materials science for the past two decades.^1–3^ Among these, nitrogenated carbon systems have emerged as particularly compelling candidates due to the synergistic effect between the high structural stability of carbon networks and the electronic modulation offered by nitrogen doping.^4–6^ Unlike pristine graphene, which lacks a natural bandgap^7,8^ nitrogen-coordinated lattices allow for the fine-tuning of the density of states near the Fermi level, making them ideal for applications in catalysis, energy storage, and nanoelectronics.^9,10^

A significant breakthrough in this field was the experimental realization of the two-dimensional nitrogenated holey graphene (2D-NHG), a stoichiometric *C*_2_ *N* atomic arrangement characterized by a well-defined distribution of nitrogen-lined pores.^11–13^ Its well-defined porous topology and semiconducting character make 2D-NHG an appealing platform for further structural engineering.^11,14^ Previous studies have shown that stacking multiple 2D-NHG layers progressively reduces the electronic bandgap as the number of layers increases.^14^ However, stacking alone does not establish covalent bonds between the layers, which retain strong in-plane covalent connectivity while interacting weakly in the out-of-plane direction. This limited out-of-plane connectivity restricts the extension of the planar network into fully connected three-dimensional (3D) architectures. Introducing covalent connections outside the original planar framework could therefore provide a route to transform the *C*_2_ *N* monolayer into 3D structures while preserving its chemically defined composition and porous character.

In this context, mechanical deformation provides a computational route for exploring the formation of covalent connections between initially stacked monolayers. Following the transformation strategy reported in ref 15, in-plane biaxial compression combined with progressive compression along the out-of-plane direction promotes structural rearrangements and the formation of covalent connections outside the original planar framework. The resulting 3D topologies can exhibit distinct electronic and optical properties.^16,17^ Importantly, different initial deformation conditions can lead to distinct relaxed structures, providing a means of exploring multiple 3D configurations from the same 2D precursor. Here, three 2D-NHG monolayers were initially stacked to provide a multilayer starting configuration for exploring the formation of 3D architectures through controlled in-plane biaxial and out-of-plane compression.

In this work, we computationally construct and characterize two distinct 3D nitrogenated holey graphene structures, designated as the 3D-NHG-α and 3D-NHG-β phases, derived from the experimentally established 2D-NHG precursor. Using first-principles calculations, we investigate their structural, energetic, electronic, and optical properties, with particular emphasis on how their bonding topologies influence their phase-dependent electronic and optical responses.

## Methodology

The 3D-NHG structures were constructed following the structural transformation protocol described in ref 15. In this approach, stacked 2D layers are subjected to in-plane biaxial compression followed by progressive compression along the out-of-plane direction, promoting the formation of covalent connections between neighboring layers.

The initial configurations were constructed by stacking three 2D-NHG monolayers arranged in an AA stacking configuration, with an initial separation of 3 Å between adjacent layers. Biaxial compressive strains ranging from 1% to 10% were considered in the *xy* plane. For each strain value, the corresponding in-plane lattice parameters were fixed and the structure was first relaxed using the Molecular Orbital PACkage (MOPAC2016),^18^ employing the PM7 semiempirical Hamiltonian. The out-of-plane lattice parameter was then progressively reduced in increments of 0.1 Å, with the atomic positions relaxed after each compression step. This procedure was continued until covalent connections between the initially stacked layers were established, as identified visually from the resulting atomic configurations, and the total energy reached a local minimum, with further compression leading to an increase in energy. Following the compression sequence, a final unconstrained structural optimization was performed, allowing both the lattice parameters and atomic positions to fully relax. Among the investigated strain values, the configurations obtained from 2% and 10% biaxial compression retained their structural integrity throughout the progressive *z*-direction compression and relaxation procedure. These configurations were selected as the 3D-NHG-α and 3D-NHG-β structures, respectively. In contrast, the remaining initial strain conditions resulted in structures exhibiting bond breaking and partial structural collapse during the relaxation sequence. These different outcomes were treated as the result of the structural screening, without assigning a specific physical stability threshold to the intermediate strain values.

The resulting 3D-NHG-α and 3D-NHG-β configurations were subsequently refined and analyzed through density functional theory (DFT) calculations using the all-electron Fritz Haber Institute Ab-Initio Molecular Simulations (FHI-AIMS)^19^ code. For these simulations, high-accuracy results were achieved by employing the standard tight basis set for all elements. Structural relaxations and primary band structure calculations were performed using the Perdew–Burke–Ernzerhof (PBE) functional within the generalized gradient approximation (GGA).^20^ To ensure high accuracy for optoelectronic properties, subsequent band structure and optical calculations were conducted using the Heyd–Scuseria–Ernzerhof (HSE06)^21^ hybrid functional. In all calculations, the van der Waals (vdW) dispersion correction was included via the Tkatchenko–Scheffler (TS) method.^22^ Based on convergence tests, a 6 × 6 × 6 Monkhorst–Pack *k*-point mesh was used for structural optimization, while a denser 12 × 12 × 12 mesh was employed for the electronic band structure and projected density of states (PDOS) calculations. During optimization, atomic positions and lattice vectors were fully optimized under periodic boundary conditions until the maximum force on each atom was below 10^–3^ eV/Å, and the total energy difference was less than 10^–6^ eV.

The structural stability of the proposed structures was assessed via cohesive energy analysis and thermodynamic simulations. The cohesive energy (*E*_coh_) was computed using the following equation:

1 $$E_{\mathrm{coh}}=\frac{1}{N_{\mathrm{total}}}\left(E_{3D-\mathrm{NHG}}-\overset{\mathrm{Ntype}}{\underset{i=1}{\sum }}N_{i}E_{i}\right)$$

where *E*_3D–NHG_ is the total energy of the structure, *N*_type_ is the different atoms types, *N*_*i*_ is the number of atomic species *i*, and *E*_*i*_ is the total energy of the isolated *i* type atom.

Due to the relatively large number of atoms in the unit cells, the dynamical stability and finite-temperature structural stability of the proposed structures were investigated within the density-functional tight-binding (DFTB) framework. The structural parameters obtained via DFTB for the proposed systems align well with the corresponding DFT calculations, as detailed in the Supporting Information (Table S1). The calculations were performed using the DFTB+ package^23^ with the mio-1-1 Slater–Koster parameters.^24^ For the phonon calculations, a 2 × 2 × 2 supercell was constructed from the relaxed unit cell, and atomic displacements of 0.01 Å were applied using the finite-displacement method implemented in the Phonopy package.^25^ The forces on the displaced supercells were evaluated using a 1 × 1 × 1 *k*-point mesh within DFTB. The phonon dispersion relations were constructed along the high-symmetry path *X*-Γ-*Y*-*L*-Γ-*Z*-*N*-Γ-*M*-*R*-Γ, with the corresponding reduced coordinates given by (0.5, 0, 0), (0, 0, 0), (0, 0.5, 0), (0.5, 0.5, 0), (0, 0, 0), (0, 0, 0.5), (0.5, 0, 0.5), (0, 0, 0), (0, 0.5, 0.5), (0.5, 0.5, 0.5), and (0, 0, 0, 0), respectively. The acoustic sum rule was applied to ensure the correct vibrational behavior at the Γ point. Finite-temperature molecular dynamics simulations were performed within the DFTB framework in the NVT ensemble for a total simulation time of 10 ps, using a 1 fs time step and a 1 × 1 × 1 *k*-point mesh. Temperature control was maintained using a Nosé–Hoover thermostat^26,27^ with a coupling strength of 3200 cm^–1^.

Spin polarized calculations were also performed in order to investigate the possible emergence of magnetic moments in the proposed structures. Both 3D-NHG configurations obtained from the strain induced compression procedure were analyzed allowing for spin polarization in the electronic structure calculations. In all cases, the results converged to a non magnetic ground state, with a total magnetic moment equal to zero, indicating the absence of magnetism in the investigated systems. Consequently, all reported results correspond to nonspin-polarized calculations.

The frequency-dependent complex dielectric function, *ε*(ω) = *ε*_1_(ω) + *i ε*_2_(ω), was evaluated within the random phase approximation (RPA)^28^ to characterize the optical response. Accordingly, the optical spectrum is governed exclusively by interband transitions, omitting quasiparticle corrections and excitonic effects. The imaginary part *ε*_2_(ω) was obtained directly from interband transitions, and the corresponding real part *ε*_1_(ω) was subsequently extracted using the Kramers–Kronig relations.^29^ From these components, the absorption coefficient *(α)* was calculated as $\alpha \left(\omega \right)=\frac{\sqrt{2}\omega }{\hbar c}{[\sqrt{\varepsilon_{1}^{2}+\varepsilon_{2}^{2}}-\varepsilon_{1}]}^{1/2}$ , where ω is the photon energy.

Structural models, atomic trajectories, and electronic density isosurfaces were processed using the Open Visualization Tool (OVITO)^30^ software. Structural parameters and the distributions of bond lengths and bond angles were computed from the optimized structures using the Atomic Simulation Environment (ASE)^31^ package.

## Results and Discussion

### Structural and Energetic Analysis

The optimized 3D-NHG structures are presented in Figure 1. For each configuration, the unit cell is displayed on the left, followed by the respective supercell crystallographic projections along the [001], [010], and [100] directions. These visual representations underscore the distinct structural features of the two phases, highlighting notable visual differences in unit cell geometry and atomic arrangement despite their shared 2D precursor.

**Figure 1:**
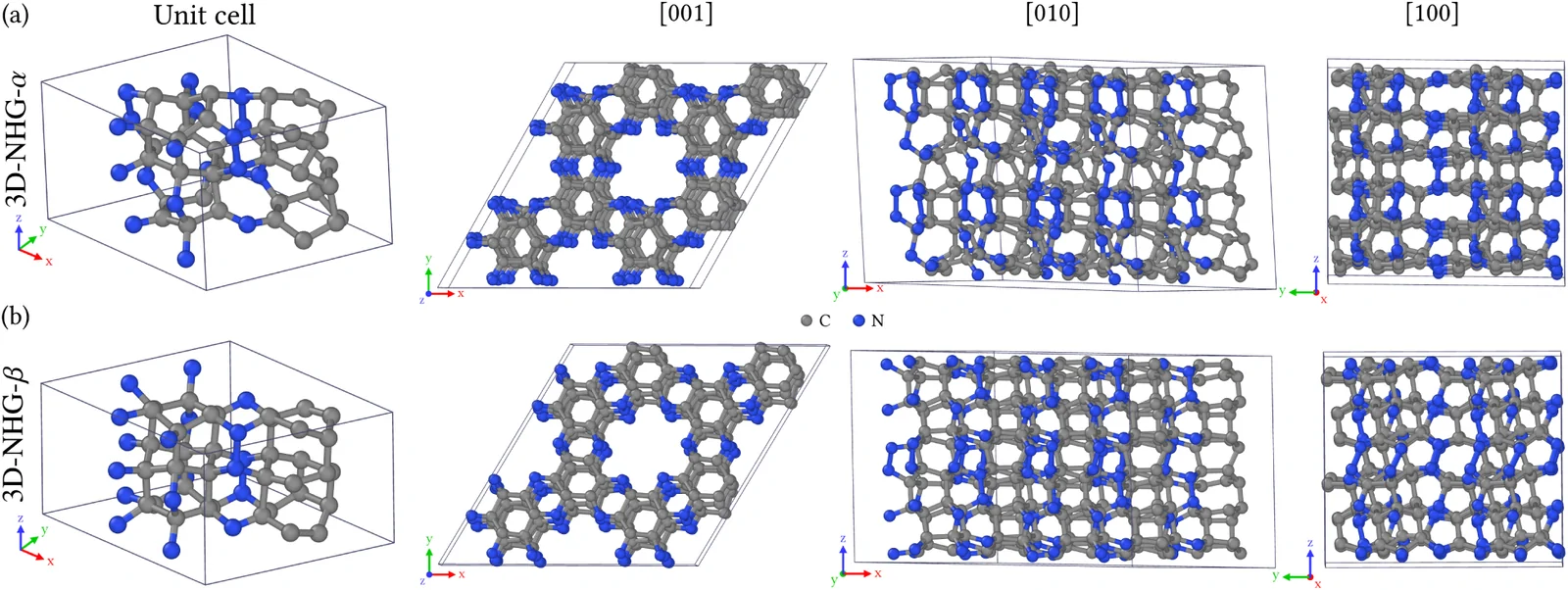
Optimized 3D-NHG structures obtained from three stacked 2D-NHG layers subjected to 2% (3D-NHG-α, top panels) and 10% (3D-NHG-β, bottom panels) initial in-plane compression. For each case, the unit cell is shown on the left, followed by the crystallographic projections of the corresponding supercell along the [001], [010], and [100] directions.

Table 1 summarizes the key structural parameters of the investigated systems, including the lattice vectors (*a*, *b*, *c*), lattice angles (α, β, γ), number of carbon (*N*_C_) and nitrogen (*N*_N_) atoms, and cohesive energy (*E*_coh_). For comparison, the corresponding data for the 2D-NHG precursor are also included. Both 3D-NHG-α and 3D-NHG-β crystallize in the triclinic *P*1 space group. The α structure exhibits slightly different in-plane lattice constants (*a* = 8.23 Å and *b* = 8.44 Å), whereas the β counterpart has nearly equal values (*a* = *b* = 8.46 Å). Despite being generated from a higher initial in-plane compression, the latter relaxes to a slightly larger in-plane cell than the former, indicating that different initial compression conditions can give rise to distinct equilibrium geometries. The out-of-plane lattice constant is 6.72 Å for 3D-NHG-α and 6.45 Å for 3D-NHG-β, indicating different interlayer separations. Furthermore, the lattice angles deviate moderately from ideal orthogonal values, with γ remaining close to 60°, reflecting the geometric influence of the hexagonal motifs inherited from the planar precursor.

**Table 1:** Calculated Lattice Parameters (*a, b, c*), Cell Angles (*α, β, γ*), Number of Carbon (*N*_C_) and Nitrogen (*N*_N_) Atoms, and Cohesive Energy (*E*_coh_) for the Optimized NHG-Based Structures

Structure	a (Å)	b (Å)	c (Å)	α (°)	β (°)	γ (°)	*N*_C_	*N*_N_	*E*_coh_ (eV/atom)
3D-NHG-α	8.23	8.44	6.72	91.0	94.9	60.7	36	18	–8.32
3D-NHG-β	8.46	8.46	6.45	90.3	92.0	59.2	36	18	–8.37
2D-NHG	8.31, 8.29^a^, 8.24^b^	8.31, 8.29^a^, 8.24^b^	-	90.1	90.3	60.0	12	6	–8.74

a Ref 32 DFT (PBE).

b Ref 11 experimental interhole distance (8.24 ± 0.96 Å).

The atomic composition of the unit cells further confirms the structural transformation process. While the primitive cell of 2D-NHG contains 12 carbon and 6 nitrogen atoms, the optimized 3D phases contain 36 carbon and 18 nitrogen atoms. This increase is consistent with the stacking of three 2D-NHG layers followed by structural reconstruction during the compression process, leading to the formation of fully connected 3D networks. The calculated cohesive energies are −8.32 eV/atom and −8.37 eV/atom for the α and β phases, respectively, compared with −8.74 eV/atom for the 2D-NHG monolayer. The slightly less negative cohesive energies of the 3D phases indicate that their formation from the 2D precursor is not energetically favorable at the level of cohesive energy, with the α and β structures being higher in energy by approximately 0.42 and 0.37 eV/atom, respectively. Nevertheless, their substantially negative cohesive energies demonstrate that both 3D structures remain energetically bound with respect to isolated atoms. Therefore, these cohesive-energy comparisons alone do not establish thermodynamic stability relative to the 2D-NHG precursor. Instead, the proposed 3D phases may be regarded as metastable structures, provided that they correspond to local minima on the potential-energy surface. Furthermore, the small cohesive-energy difference of approximately 50 meV/atom indicates that the two phases possess similar energetic stability within the present level of theory.

The local bonding environments in the two 3D-NHG structures were further analyzed through the statistical distributions of bond lengths and bond angles, as shown in Figure 2. The overall bond-length distributions illustrated in Figure 2a indicate that both phases exhibit similar ranges centered around typical C–C and C–N bonding distances. However, the 3D-NHG-α configuration exhibits a slightly broader distribution, suggesting a larger variability in local bonding environments. A comparable trend is observed for the bond-angle distributions in Figure 2b, where both histograms exhibit broad peaks centered around 110°, indicating similar local angular environments in the two structures. The decomposition by atom pairs presented in Figure 2c reveals clear differences among C–C, C–N, and N–N bonds, with C–N bonds generally exhibiting shorter average lengths due to the smaller atomic radius and higher electronegativity of nitrogen. The bond angle distributions grouped by atomic triplets, shown in Figure 2d, are centered within a similar angular range for both structures. However, the 3D-NHG-α phase exhibits slightly broader distributions, indicating a larger spread in the local angular environments. Overall, these results suggest that the two phases retain comparable fundamental bonding motifs, while the α structure accommodates a somewhat higher degree of local geometrical distortion.

**Figure 2:**
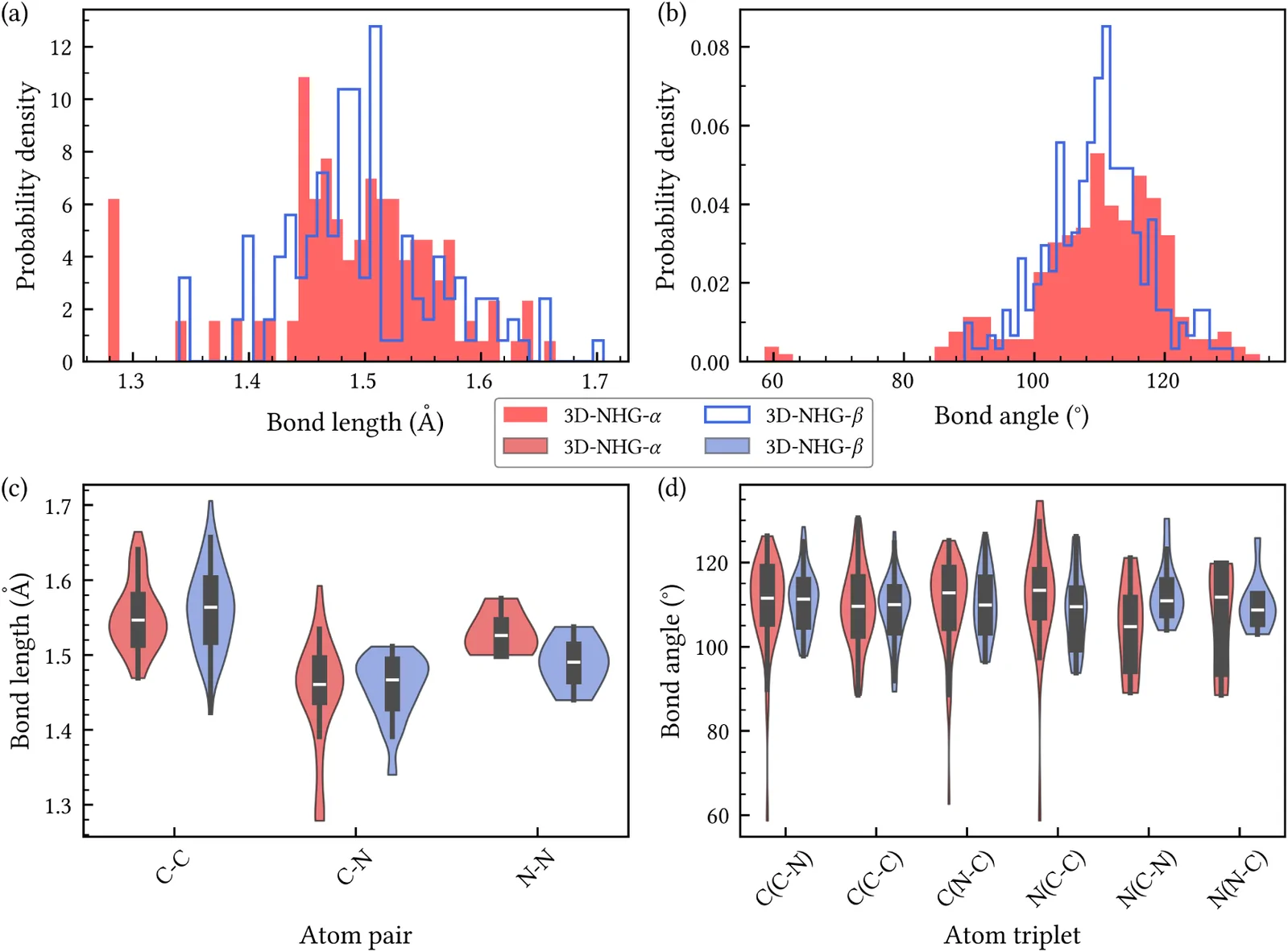
Bond-length and bond-angle distributions for the 3D-NHG-α and 3D-NHG-β phases. (a, b) Overall probability density of bond lengths and bond angles. (c, d) Distributions grouped by atom pairs and atom triplets. Violin plots show the full distributions with embedded boxplots indicating the median and interquartile range.

To further characterize the bonding nature of the proposed structures, the electron localization function (ELF) was analyzed for both configurations, as shown in Figure 3. The ELF quantifies the degree of electron localization, where values approaching 1 correspond to strongly localized electrons, as typically found in covalent bonds or lone-pair regions. Conversely, a value of 0.5 represents a homogeneous electron-gas-like distribution, while values approaching zero indicate regions of minimal localization. The resulting ELF maps reveal similar patterns of electron localization for the C–C and N–N connections in both the 3D-NHG-α and 3D-NHG-β configurations. In particular, pronounced electron localization is observed along the C–C connections extending outside the original plane of the stacked 2D-NHG sheets, with ELF values approaching unity. The corresponding N–N connections also exhibit substantial electron localization, with ELF values of approximately 0.8, consistent with localized shared-electron bonding. These features provide strong evidence for the covalent character of the newly formed out-of-plane connections, rather than interactions arising solely from weak interlayer forces. Despite the distinct atomic rearrangements and geometries of the two systems, both exhibit strongly localized electron density along bonds extending outside the original planar framework. This finding demonstrates that different structural rearrangements of the stacked 2D-NHG precursor can lead to 3D architectures interconnected by covalent bonds.

**Figure 3:**
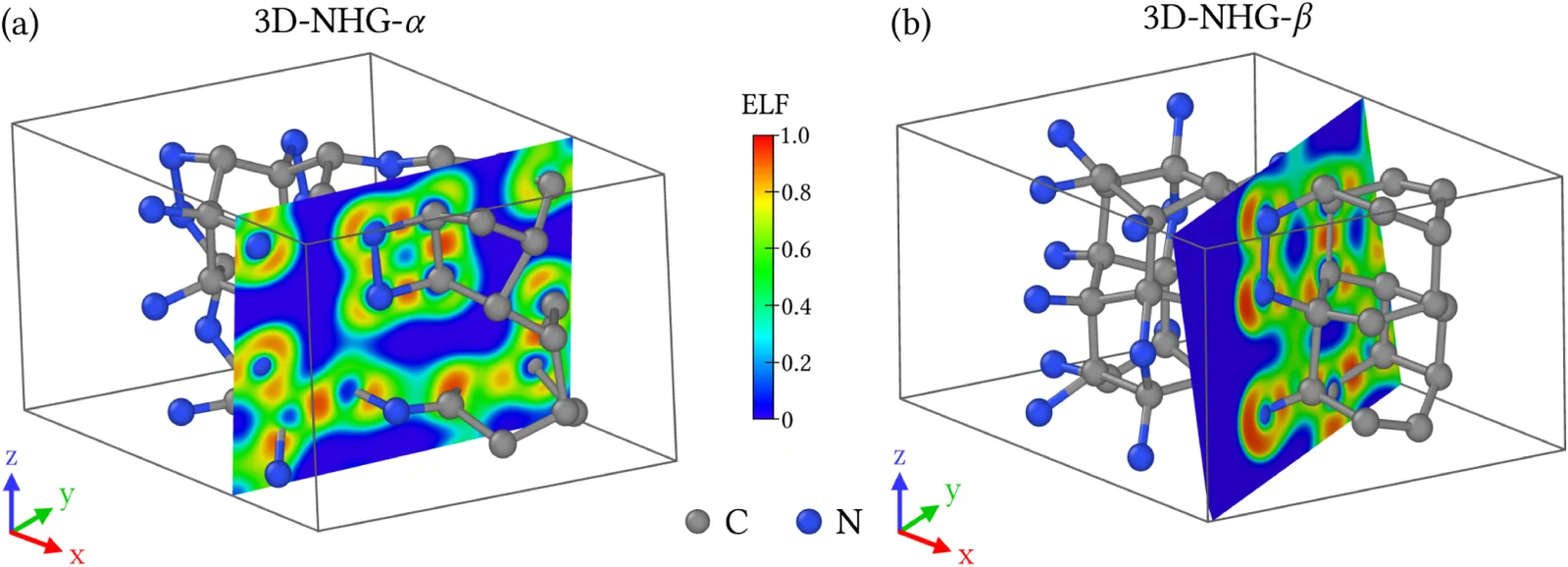
Electron localization function (ELF) maps for (a) 3D-NHG-α and (b) 3D-NHG-β structures. The colored surfaces show the ELF distribution on planes crossing the out-of-plane covalent bonding regions. The color scale ranges from 0 (blue) to 1 (red), with higher values indicating stronger electron localization.

### Dynamical and Structural Stability

The phonon dispersions of the 3D-NHG-α and 3D-NHG-β phases are shown in Figure 4. Both structures exhibit no imaginary phonon frequencies throughout the Brillouin zone, indicating dynamical stability within the DFTB framework. The two phases nevertheless display distinct vibrational spectra. The phonon modes of the 3D-NHG-β phase extend up to approximately 50 THz, whereas the 3D-NHG-α phase exhibits vibrational modes reaching higher frequencies, up to approximately 57 THz. This difference reflects the distinct local bonding environments and structural arrangements of the two phases, which lead to different vibrational frequency ranges. In the 3D-NHG-β phase, relatively flat and well-separated modes are observed above approximately 45 THz, whereas similar isolated features in the 3D-NHG-α phase appear only at higher frequencies, above approximately 50 THz. These high-frequency modes are characteristic of localized vibrations associated with specific atomic environments rather than collective lattice motions. The presence of these isolated high-frequency features in both phases further highlights the heterogeneous nature of their 3D bonding networks.

**Figure 4:**
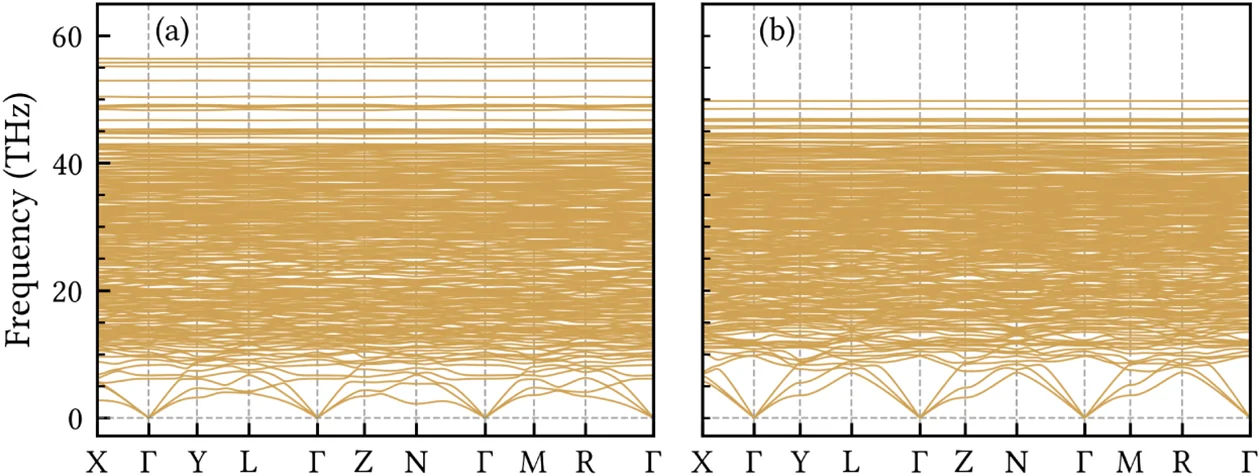
DFTB phonon dispersion relations for (a) 3D-NHG-α and (b) 3D-NHG-β phases.

The structural stability of the proposed systems is accessed by the DFTB-based molecular dynamics results shown in Figure 5, which depict the evolution of the total energy per atom at 300 and 1000 K. Throughout the entire simulation time, the energy fluctuates within a narrow range around a nearly constant average value, with no discontinuities associated with bond breaking or structural degradation. The final atomic configurations extracted from the trajectories, shown in the inset images, further confirm that both structures preserve their structural integrity under the investigated temperature conditions. Although thermal vibrations and minor local distortions are observed, no lattice collapse or large-scale reconstruction occurs relative to the optimized geometries shown in Figure 1.

**Figure 5:**
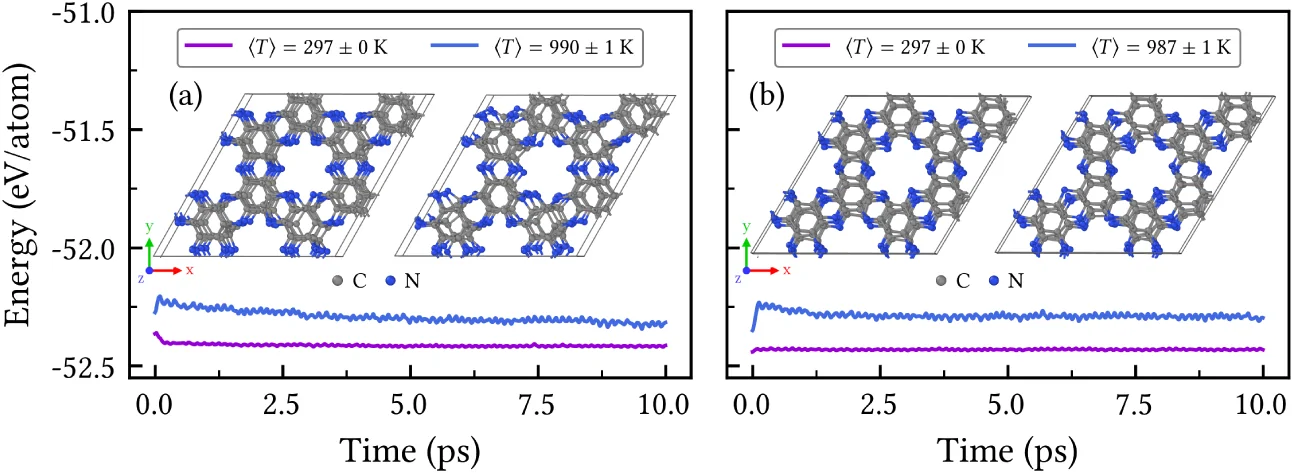
Energy fluctuations during DFTB-based molecular dynamics simulations at 300 K (purple) and 1000 K (blue) for (a) 3D-NHG-α and (b) 3D-NHG-β phases. Final structural snapshots at each investigated temperature are shown in the insets.

### Electronic Properties

The electronic properties of the proposed structures are presented in Figure 6. Panels (a) and (b) display the HSE06 electronic band structures and corresponding projected density of states (PDOS) for the 3D-NHG-α and 3D-NHG-β phases, respectively. The spatial charge distributions associated with the valence-band maximum (VBM) and conduction-band minimum (CBM) are shown in panels (c) and (d). The corresponding PBE band structures and PDOS are provided in the (Supporting Information Figure S1).

**Figure 6:**
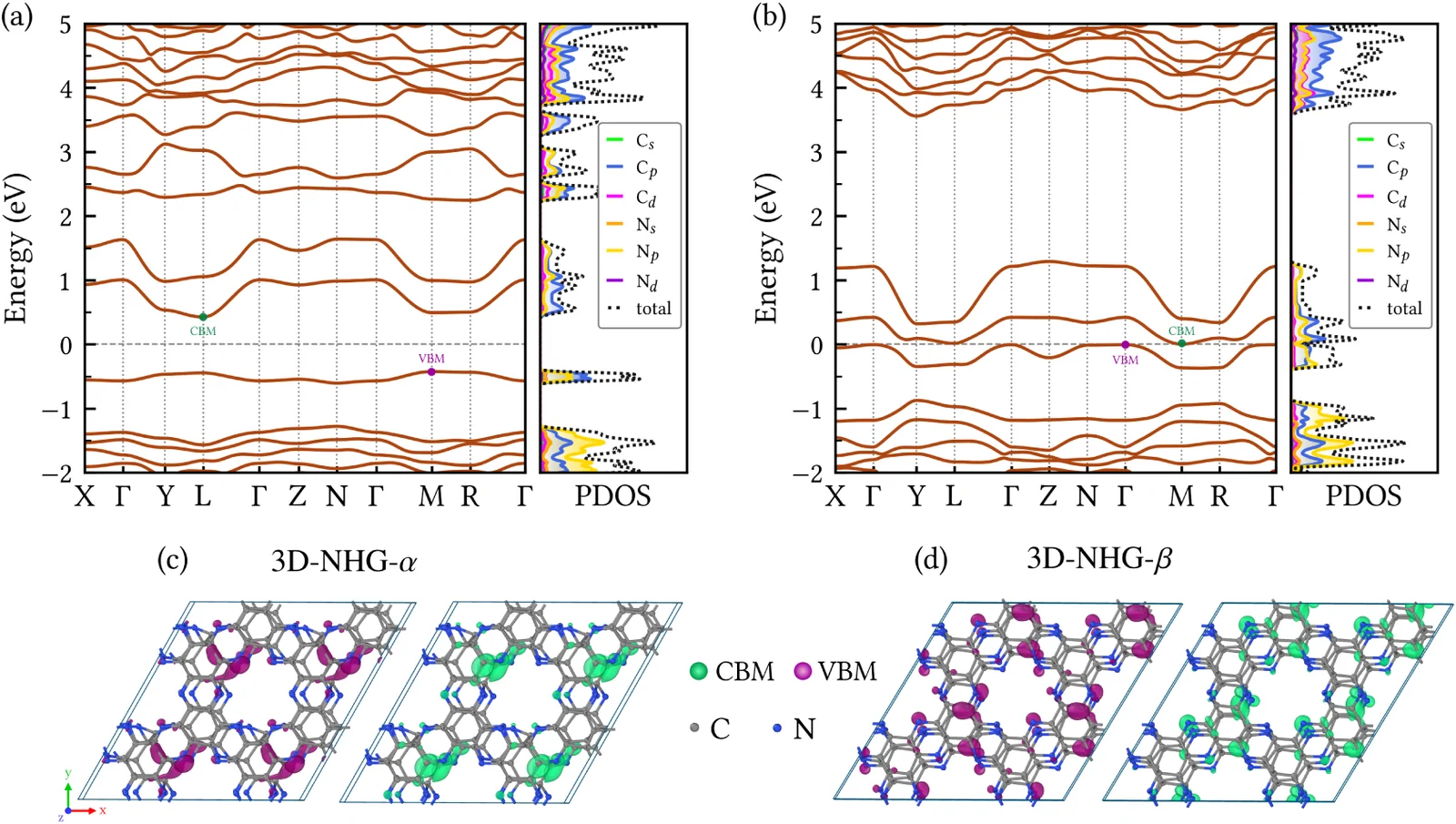
Electronic band structure and projected density of states (PDOS) for (a) 3D-NHG-α and (b) 3D-NHG-β structures, calculated at the HSE level. A Gaussian broadening of 25 meV was applied to the PDOS, and the horizontal gray dashed lines indicate the Fermi level. Isosurface representations of the valence-band maximum (VBM) and conduction-band minimum (CBM) for each structure are shown in (c) and (d), respectively. The isosurface level is set to ±0.05 e Å^–3^.

The 3D-NHG-α phase exhibits an indirect bandgap of 0.87 eV, with the VBM and CBM located at the M and L high-symmetry points, respectively. In contrast, the electronic structure of the 3D-NHG-β phase exhibits a zero-gap semiconducting character. The valence and conduction bands touch the Fermi level at distinct high-symmetry points, namely Γ and M, respectively, giving rise to a zero bandgap. These electronic properties differ markedly from those of the parent 2D-NHG monolayer, whose HSE06 electronic structure and PDOS are presented in the (Supporting Information Figure S3). The calculated HSE06 bandgap of 2.44 eV is consistent with previous theoretical reports of 2.34 eV^14^ and 2.41 eV.^33^ Experimentally, an optical bandgap of approximately 1.96 eV has been reported for the 2D-NHG.^11^ Previous calculations have also shown that the bandgap decreases progressively with increasing numbers of stacked 2D-NHG layers, with HSE06 values of 2.34, 2.12, 2.02, and 1.94 eV reported for the monolayer, bilayer, trilayer, and bulk systems, respectively.^14^ This trend indicates that interlayer coupling associated with stacking already modifies the electronic structure, even without the formation of new covalent connections between the layers. In the present structures, however, the reduction of the bandgap is substantially more pronounced. This pronounced reduction is consistent with the formation of the out-of-plane covalent connections identified by the ELF analysis in Figure 3, which introduces additional electronic coupling pathways within the 3D system. These results demonstrate that the formation of the 3D covalent network substantially modifies the electronic structure, transforming the wide-bandgap semiconducting 2D precursor into a narrow-gap semiconductor (3D-NHG-α) and a zero-gap semiconductor (3D-NHG-β).

Analysis of the frontier bands provides additional insight into the electronic structure. As shown in Figure 6a, the valence- and conduction-band edges of 3D-NHG-α exhibit moderate dispersion along several high-symmetry directions, indicating that the corresponding electronic states are not strongly localized. In contrast, the bands closest to the Fermi level in the 3D-NHG-β configuration display a more pronounced dispersion, particularly along selected directions of the Brillouin zone, consistent with its zero-gap electronic structure.

The PDOS shown in the right panels of Figure 6a–b provides insight into the orbital origin of the electronic states near the Fermi level. In both phases, these states are dominated by the *p* orbitals of both carbon and nitrogen atoms, with only negligible contributions from the remaining orbitals. Further insight into the electronic structure is obtained from the spatial distributions of the VBM and CBM states shown in Figure 6c–d. In the 3D-NHG-α phase, the band-edge states are concentrated in specific regions of the structure while remaining sufficiently extended to account for the observed band dispersion. In contrast, the corresponding states in 3D-NHG-β are distributed more uniformly throughout the framework, consistent with the stronger band dispersion and zero-gap semiconducting character. These electronic characteristics are closely related to the structural transformation from the planar 2D-NHG precursor to the 3D covalent networks. The formation of interlayer covalent bonds modifies the connectivity of the original lattice and changes the orbital interactions between neighboring atoms, leading to a substantial reconstruction of the electronic band structure. Despite these structural changes, the PDOS reveals that the electronic states near the Fermi level remain dominated by carbon and nitrogen *p* orbitals in both phases.

### Optical Properties

The optical response of the proposed structures at the HSE06 level is presented in Figure 7, where the real and imaginary parts of the dielectric function, together with the absorption coefficient, are shown for the three crystallographic directions. The spectra are displayed for the 3D-NHG-α (left panels) and the 3D-NHG-β phase (right panels), allowing a direct comparison of their optical behavior across a broad photon energy range. Enlarged views of the visible spectral region are provided in Figure S2 of the Supporting Information, while the corresponding HSE06 optical spectra of the parent 2D-NHG are shown in Figure S4.

**Figure 7:**
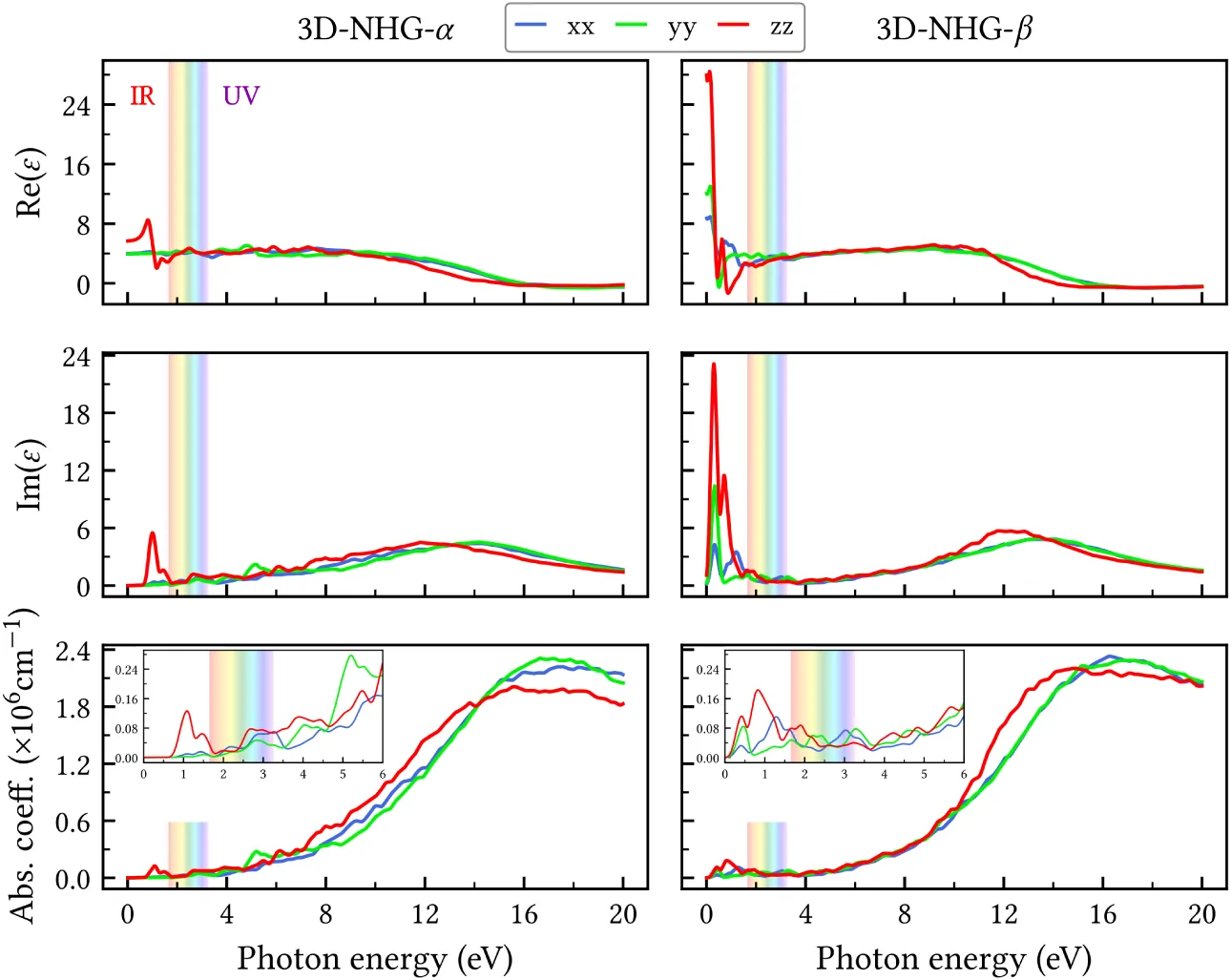
Optical properties for 3D-NHG-α (left panels) and 3D-NHG-β (right panels) structures obtained from HSE calculations. The real (Re­(*ε*)) and imaginary (Im­(*ε*)) parts of the dielectric function and the absorption coefficient are plotted as a function of photon energy for the xx, yy, and zz components of the dielectric tensor. The shaded region represents the visible range, highlighting the relevant spectral window for optoelectronics applications, while the insets magnify the absorption coefficient below 6 eV.

In the low-energy region, corresponding to the infrared (IR) regime, the 3D-NHG-α phase exhibits relatively smooth dielectric behavior, with the imaginary part of the dielectric function remaining close to zero below the calculated absorption onset of approximately 0.87 eV, consistent with its finite electronic bandgap. For the 3D-NHG-β phase, the low-energy optical response cannot be fully interpreted within the present interband-only treatment. In particular, the calculated response does not include possible intraband (Drude) contributions, which may become relevant for a zero-gap semiconductor, particularly at finite temperature or in the presence of free carriers. Therefore, the calculated low-energy dielectric response does not represent the complete optical response of this phase.

Within the visible and ultraviolet (UV) spectral regions, the real and imaginary parts of the dielectric function exhibit broadly similar profiles for both structures. For the 3D-NHG-α phase, the real part remains approximately constant up to 8 eV, after which it gradually decreases and approaches zero at photon energies above 16 eV. A similar behavior is observed for the 3D-NHG-β phase, although the decrease in the real part begins at a slightly higher energy, around 10 eV, with Re­(*ε*) also approaching zero above 16 eV. The imaginary part, in contrast, increases with photon energy for both structures, reaching broad maxima near 12 eV, followed by a gradual decrease at higher energies. These features reflect the evolution of interband optical transitions over the visible and UV spectral ranges. The absorption spectra shown in the bottom panels of Figure 7 follow the same overall trend in this energy regime. The absorption coefficient begins to increase near the visible range, reaching broad maxima around 17 eV before gradually decreasing at higher photon energies. As shown in the insets of the bottom panels, the absorption coefficient remains negligible throughout the visible range, with only small differences among the polarization directions up to 6 eV. At higher photon energies, the differences between the in-plane and out-of-plane components become more pronounced. The degree of in-plane anisotropy also differs between the two phases, being more pronounced in the 3D-NHG-α structure.

## Conclusions

In summary, we have introduced two novel three-dimensional nitrogenated holey graphene (3D-NHG) structures, designated as the α and β phases, obtained through a controlled computational compression protocol of stacked 2D-NHG monolayers. Phonon calculations and density-functional tight-binding molecular dynamics simulations indicate that both configurations are dynamically stable and preserve their structural integrity under the investigated finite-temperature conditions of approximately 1000 K.

Electronic structure calculations within the density functional theory framework reveal distinct electronic behaviors for the two architectures. The 3D-NHG-α phase is a semiconductor with an indirect HSE06 bandgap of 0.87 eV, whereas the 3D-NHG-β phase exhibits a zero-gap semiconducting character, with the valence and conduction bands touching the Fermi level at different high-symmetry points. In both cases, the electronic states near the Fermi level are dominated by the *p* orbitals of carbon and nitrogen atoms. Optical calculations further demonstrate a strong absorption in the ultraviolet region, with negligible absorption throughout the visible range. Overall, these findings demonstrate that the transformation from 2D to 3D carbon nitride frameworks substantially modifies their electronic and optical properties, providing a platform for exploring structure–property relationships in future optoelectronic materials.

## Supplementary Material





## Notes

Crystallographic Information Files (CIFs) for the optimized 3D-NHG structures (ZIP)
Additional structural, electronic, and optical properties are provided, including a comparison of the structural parameters obtained using DFTB and DFT, PBE band structures and PDOS for the 3D-NHG phases, enlarged optical spectra in the visible range, and HSE06 electronic and optical properties of the parent 2D-NHG structure (PDF)
