# Supplementary material for: Computational Design of Three-Dimensional Nitrogenated Holey Graphene Structures with Phase-Dependent Optoelectronic Properties
Source: ACS Omega. 2026 Sep 25;11(39):60259–67. doi: 10.1021/acsomega.6c07967 (PMC13647830; doi:10.1021/acsomega.6c07967)
Supplement: Supplementary file 1 [file ao6c07967_si_002.pdf]

Supplementary Material for Computational design of three-dimensional nitrogenated holey graphene structures with phase-dependent optoelectronic properties

Jhionathan de Lima,[†,‡] Raphael M. Tromer,[¶,§] Luiz F. C. Pereira,[‖] Mauro S. Ferreira,[⊥,#] and Cristiano F. Woellner[*,†,‡]

†*Department of Physics, Federal University of Parana, UFPR, Curitiba, PR, 81531-980, Brazil.*

‡*Interdisciplinary Center for Science, Technology, and Innovation (CICTI), Federal University of Parana, UFPR, Curitiba, PR, 81530-000, Brazil.*

¶*Institute of Physics, University of Brasília, Brasília, DF, Brazil*

§*Center for Computational Engineering and Sciences, State University of Campinas, Campinas, SP, 13083-970, Brazil*

‖*Physics Department, Federal University of Pernambuco Recife, PE, 50670-901, Brazil.*

⊥*School of Physics, Trinity College Dublin, Dublin 2, Ireland.*

#*Centre for Research on Adaptive Nanostructures and Nanodevices (CRANN) and Advanced Materials and Bioengineering Research (AMBER) Centre, Trinity College Dublin, Dublin 2, Ireland .*

E-mail: woellner@ufpr.br

This Supplementary Material presents additional structural, electronic, and optical characterization of the investigated nitrogenated holey graphene structures. First, a quantitative comparison of structural parameters obtained via DFT and DFTB is provided to demonstrate the accuracy and consistency of the DFTB structural description. The text then includes the PBE band structures and projected density of states (PDOS) for the 3D-NHG phases, a magnified view of their optical spectra in the visible energy range, and the HSE06 electronic band structure, PDOS, and optical response of the parent 2D-NHG monolayer. These complementary results provide further support for the discussion presented in the main manuscript and facilitate the comparison between the 2D precursor and the proposed 3D architectures.

Structural comparison between DFT and DFTB

To assess the consistency of the structural description obtained with DFTB, the optimized structural parameters were compared with the corresponding DFT results for both 3D-NHG phases. Table S1 summarizes the lattice parameters, cell angles, average bond distance, and volumetric density obtained using the two methods.

Overall, the DFTB results show good agreement with the corresponding DFT structural parameters. For 3D-NHG-α, the lattice parameters differ by at most 0.07 Å, corresponding to relative deviations below 1.1 %. The cell angles differ by at most 0.1°, while the average bond distance is identical at the reported precision. The volumetric density differs by only 0.01 g/cm^3, corresponding to a relative deviation of approximately 0.4 %. For 3D-NHG-β, the lattice parameters differ by at most 0.03 Å, corresponding to relative deviations below 0.5 %. The maximum angular deviation is 0.3°, while the average bond distance differs by only 0.01 Å, corresponding to a relative deviation of approximately 0.7 %. The density differs by 0.02 g/cm^3, corresponding to a relative deviation of approximately 0.7 %. These results indicate that DFTB provides a consistent structural description of both 3D-NHG phases,

supporting its use for the phonon and finite-temperature calculations performed in this work.

Table S1: Comparison of the optimized lattice parameters (a, b, c), cell angles (α, β, γ), average bond distance (R_{ave}), and volumetric density (ρ) obtained from DFT and DFTB calculations for the 3D-NHG structures.

Structure	Method	a (Å)	b (Å)	c (Å)	α(°)	β(°)	γ(°)	R_{ave} (Å)	ρ (g/cm^3)
3D-NHG-α	DFT	8.23	8.44	6.72	91.0	94.9	60.7	1.51	2.80
	DFTB	8.24	8.51	6.64	91.1	94.9	60.7	1.51	2.81
3D-NHG-β	DFT	8.46	8.46	6.45	90.3	92.0	59.2	1.51	2.87
	DFTB	8.49	8.49	6.44	90.2	92.0	58.9	1.52	2.85

Electronic properties of the 3D-NHG phases

Figure S1 presents the electronic band structures and projected density of states (PDOS) for the (a) 3D-NHG-α and (b) 3D-NHG-β structures calculated using the PBE exchange-correlation functional. At this level of theory, the 3D-NHG-α phase exhibits an indirect bandgap of 0.16 eV, with the valence-band maximum (VBM) and conduction-band minimum (CBM) occurring at the M and L high-symmetry points, respectively. In contrast, the 3D-NHG-β phase exhibits a metallic character, with the valence and conduction bands crossing the Fermi level and a finite density of states at the Fermi energy. These PBE results are provided for comparison with the HSE06 calculations presented in the main text.

Optical properties of the 3D-NHG phases

Figure S2 presents an enlarged view of the optical response of the 3D-NHG-α (left panels) and 3D-NHG-β (right panels) phases within the visible spectral range (from 1.65 eV to 3.26 eV). This representation highlights the weaker spectral features that are less apparent in the full-energy-range plots shown in the main text, allowing a clearer comparison of the anisotropy in the dielectric function and absorption coefficient strictly within this visible window, which constitutes the range of interest for most optoelectronic applications.

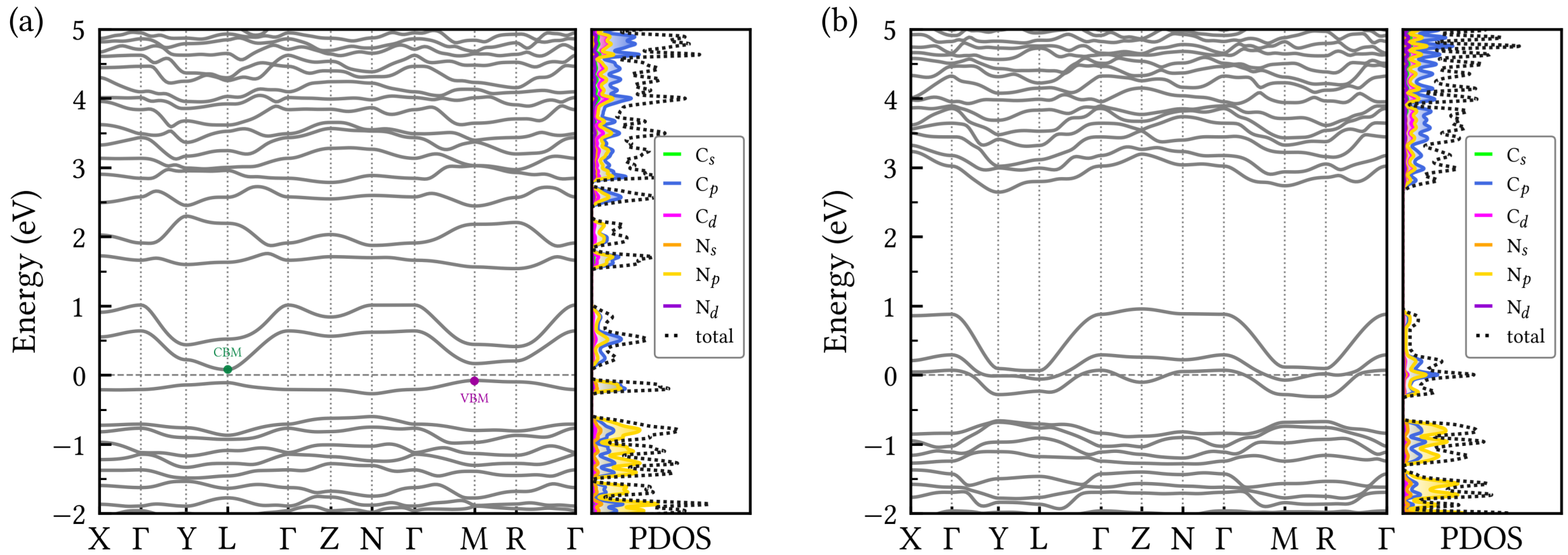

Figure S1: Electronic band structure and projected density of states (PDOS) for (a) 3D-NHG-α and (b) 3D-NHG-β structures, calculated at the PBE level. A Gaussian broadening of 25 meV was applied to the PDOS, and the horizontal gray dashed lines indicate the Fermi level. The acronyms VBM and CBM stand for valence-band maximum and conduction-band minimum, respectively.

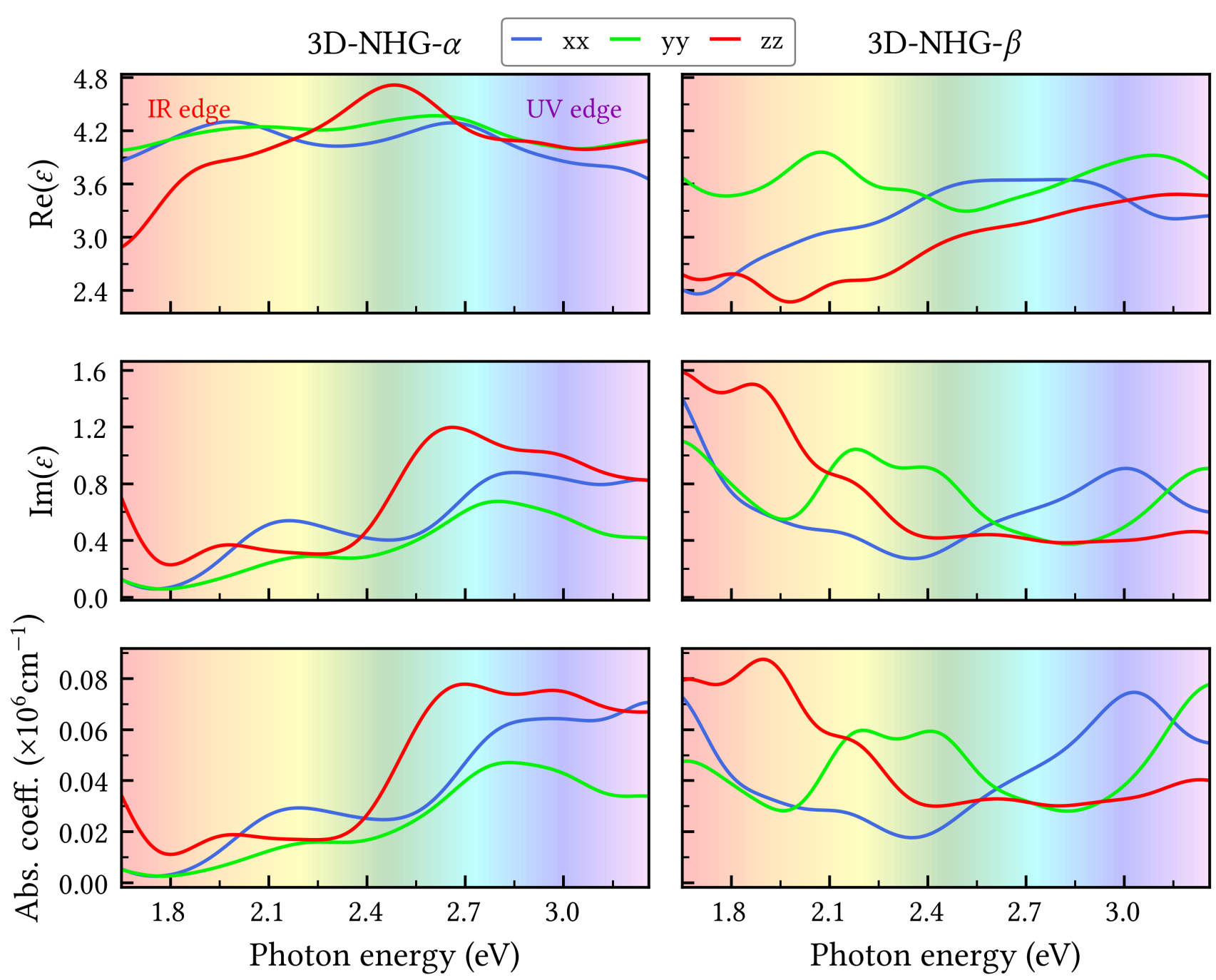

Figure S2: Optical properties for 3D-NHG-α (left panels) and 3D-NHG-β (right panels) structures obtained from HSE calculations over the visible spectral region. The real (Re(ε)) and imaginary (Im(ε)) parts of the dielectric function and the absorption coefficient are plotted as a function of photon energy for the xx, yy, and zz components of the dielectric tensor.

Electronic and optical properties of the 2D-NHG

Figure S3 presents the HSE06 electronic band structure and projected density of states (PDOS) of the parent 2D-NHG structure. The calculated band structure exhibits a direct bandgap of 2.44 eV at the Γ point, in good agreement with previous HSE06 calculations reported in the literature (2.34 eV and 2.41 eV from Refs.,[1,2] respectively), while PBE calculations report a value of approximately 1.70 eV.[1,3] The states near the band edges are predominantly composed of carbon and nitrogen p orbitals, while the contributions from s and d orbitals are comparatively small. These results provide a consistent reference for comparison with the electronic structures of the proposed 3D-NHG phases discussed in the main text.

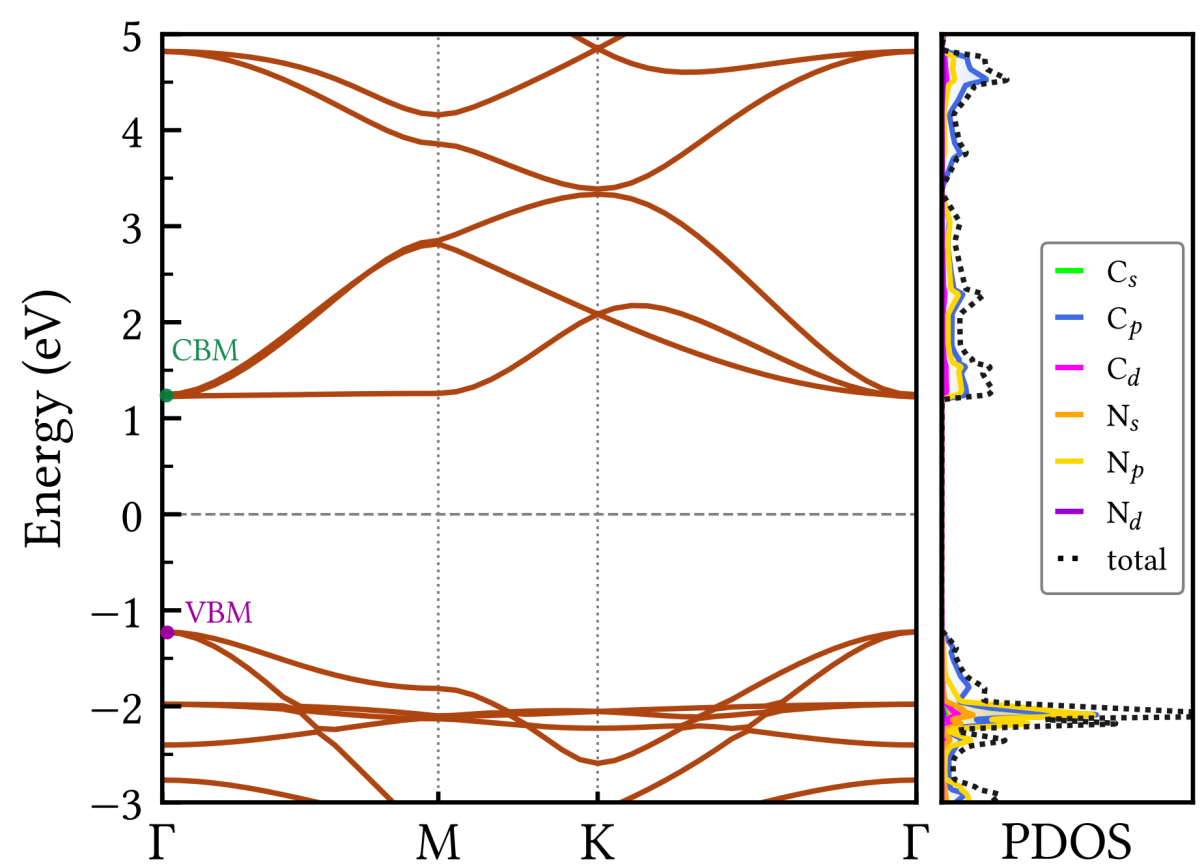

Figure S3: Electronic band structure and projected density of states (PDOS) for the 2D-NHG calculated at the HSE level. A Gaussian broadening of 25 meV was applied to the PDOS, and the horizontal gray dashed lines indicate the Fermi level. The acronyms VBM and CBM stand for valence-band maximum and conduction-band minimum, respectively.

Figure S4 shows the optical response of the 2D-NHG structure at the HSE level, including the the real and imaginary parts of the dielectric function, as well as the absorption coefficient. The isotropic optical in-plane behavior is confirmed by the overleap of the spectra for the xx and yy directions. The calculated absorption onset is 2.43 eV, which is closed to our calculated electronic bandgap of 2.44 eV at the same level of theory.

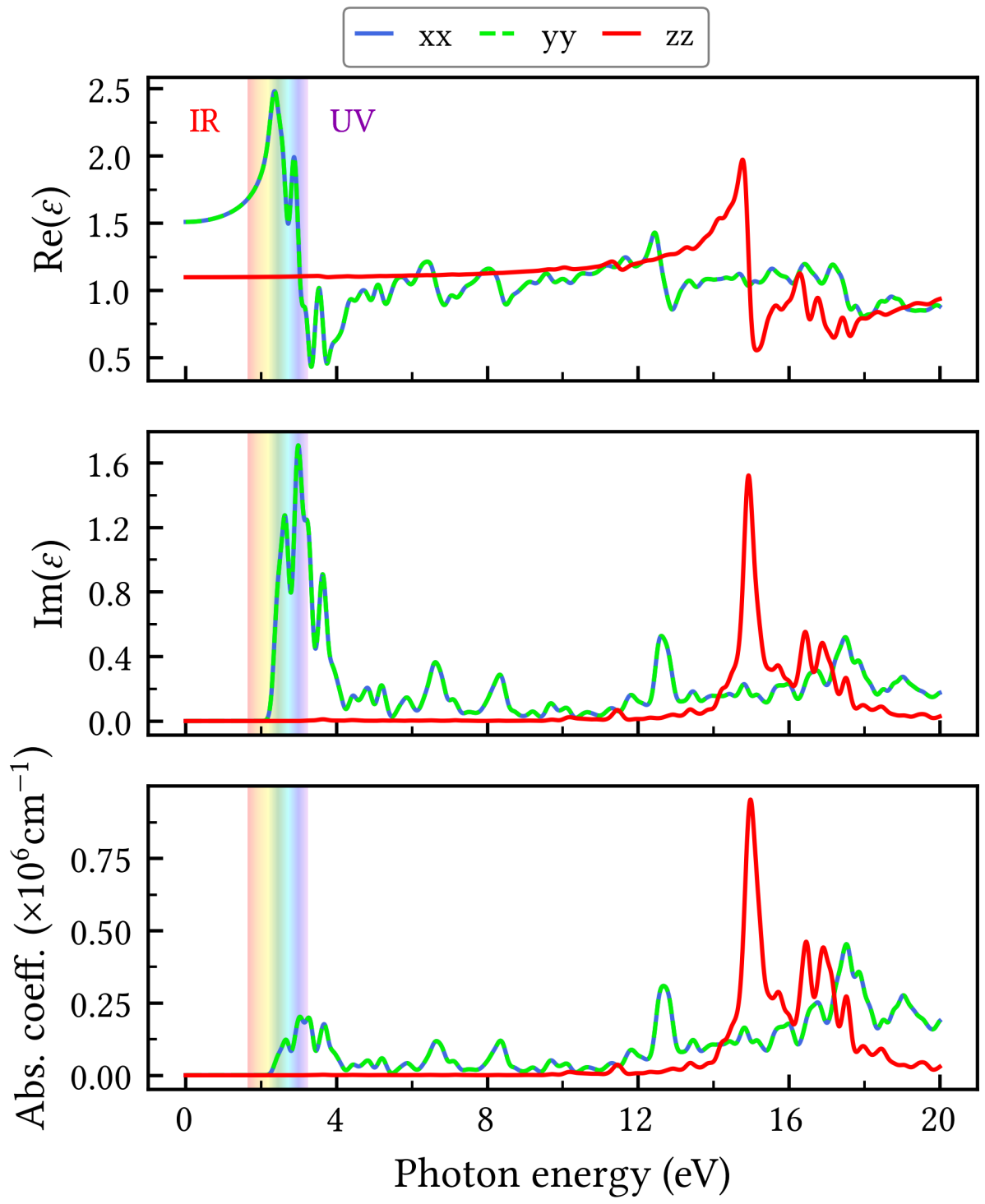

Figure S4: Optical properties for the 2D-NHG obtained from HSE calculations. The real (Re(ε)) and imaginary (Im(ε)) parts of the dielectric function and the absorption coefficient are plotted as a function of photon energy for the xx, yy, and zz components of the dielectric tensor. The shaded region represents the visible range, highlighting the relevant spectral window for optoelectronics applications.

References

(1) Xu, C.-Y.; Dong, H.-K.; Shi, L.-B. First principles investigation of nitrogenated holey graphene. *Physica E: Low-dimensional Systems and Nanostructures* **2018**, *98*, 135–139.

(2) Liu, Y.; Ye, C.; Zhao, H.; Lin, K.; Cao, X.; Ai, Y. Si-Doped Nitrogenated Holey Graphene (C_2N) as a Promising Gas Sensor for O-Containing Volatile Organic Compounds (VOCs) and Ammonia. *Crystals* **2023**, *13*, 816.

(3) Mahmood, J.; Lee, E. K.; Jung, M.; Shin, D.; Jeon, I.-Y.; Jung, S.-M.; Choi, H.-J.; Seo, J.-M.; Bae, S.-Y.; Sohn, S.-D. et al. Nitrogenated holey two-dimensional structures. *Nature Communications* **2015**, *6*.
